# Application of Constitutive Models and Machine Learning Models to Predict the Elevated Temperature Flow Behavior of TiAl Alloy

**DOI:** 10.3390/ma16144987

**Published:** 2023-07-13

**Authors:** Rui Zhao, Jianchao He, Hao Tian, Yongjuan Jing, Jie Xiong

**Affiliations:** 1 Institute of Special Environment Physical Sciences, Harbin Institute of Technology, Shenzhen 518055, China; zhaorui2020@hit.edu.cn; 2 School of Materials Science and Engineering, Harbin Institute of Technology, Shenzhen 518055, China; tianhao19961207@163.com; 3 AVIC Beijing Aeronautical Manufacturing Technology Research Institute, Beijing 100024, China; jingyongjuan1982@126.com; 4 State Key Laboratory of Advanced Welding and Joining, Harbin Institute of Technology, Harbin 150001, China

**Keywords:** TiAl alloy, hot-deformation behavior, data-driven model, generalization capability

## Abstract

The hot deformation behaviors of a Ti46Al2Cr2Nb alloy were investigated at strain rates of 0.001–0.1 s^−1^ and temperatures of 910–1060 °C. Under given deformation conditions, the activation energy of the TiAl alloy could be estimated as 319 kJ/mol. The experimental results were predicted by different predictive models including three constitutive models and three data-driven models. The most accurate data-driven model and constitutive model were an artificial neural network (ANN) and an Arrhenius type strain-compensated Sellars (SCS) model, respectively. In addition, the generalization capability of ANN model and SCS model was examined under different deformation conditions. Under known deformation conditions, the ANN model could accurately predict the flow stress of TiAl alloys at interpolated and extrapolated strains with a coefficient of determination (*R*^2^) greater than 0.98, while the *R*^2^ value of the SCS model was smaller than 0.5 at extrapolated strains. However, both ANN and SCS models performed poorly under new deformation conditions. A hybrid model based on the SCS model and ANN predictions was shown to have a wider generalization capability. The present work provides a comprehensive study on how to choose a predictive model for the flow stress of TiAl alloys under different conditions.

## 1. Introduction

TiAl based alloys are elevated-temperature structural materials with a high specific strength, great specific modulus, good creep resistance, outstanding elevated temperature strength, and great oxidation resistance [1,2,3,4]. TiAl alloys are potential materials in automotive and aerospace industries due to the aforementioned excellent properties [5,6]. However, the hot workability of TiAl alloys is limited, which inhibits them from being broadly employed for desirable applications [7,8,9]. Understanding the hot-deformation behaviors of TiAl alloys is critical to define optimal thermomechanical processing conditions within the limitation of workability. Therefore, many efforts have been put into constructing predictive models for the hot-deformation behaviors of TiAl alloys [7,10,11,12,13].

In the past decades, the hot-deformation behaviors of TiAl alloys and other alloys at elevated temperatures have been examined, and predictive constitutive models have been developed on the basis of these investigations [14,15]. For instance, Kong et al. [11] employed the Arrhenius type constitutive model to predict the peak stress of Ti-48Al-2Cr-4Nb-0.2Y alloys under deformation temperatures of 1100–1250 °C at strain rates of 0.01–1 s^−1^, which suggested the optimal processing temperature and strain rate in the range of 1200–1230 °C and 0.01–0.05 s^−1^, respectively. Cheng et al. [10] proposed a constitutive model involving different softening mechanisms, and the resulting predictive model could give an accurate estimate of the flow stress of a high-Nb-containing TiAl alloy. Sun et al. [16] examined and described the hot-deformation behaviors of powder metallurgy (PM) TiAl alloys with the Arrhenius type model. They found that the PM TiAl alloy exhibited some flow instability at strain rates higher than 0.01 s^−1^, indicating that the processing strain rate should be slower than 0.01 s^−1^.

Recently, more and more date-driven models such as artificial neural networks (ANNs) [17,18,19], support vector machines (SVMs) [20], random forests (RFs) [21], and Gaussian process regressors (GPRs) [22] have been developed to predict the hot-deformation behaviors of alloys with the development of machine learning techniques. Ge et al. [17] utilized the ANN model and Arrhenius type model to predict the hot-deformation behavior of a high-Nb-containing TiAl alloy with *β* + *γ* phases. Their results revealed that ANN models were more accurate than Arrhenius type models in predicting the hot-deformation behaviors of TiAl alloys. However, most of the predictive models proposed have been based on the Arrhenius type model and ANN model. The performance of other constitutive models and data-driven models have rarely been reported. A comprehensive comparison of different predictive models is needed. In addition, the generalization capabilities of the predictive models have only been examined under known deformation conditions, and the performance of predictive models under unknown deformation conditions should be investigated as well. Furthermore, the combination of ML model and mechanism-based constitutive models has not been considered in previous work.

In the present work, the hot-deformation behaviors of TiAl alloys at strain rates of 0.001–0.1 s^−1^ and temperatures of 910–1060 °C were examined. The experimental results were predicted via three constitutive models and three machine learning (ML)-based models. The prediction accuracies on the training data of six predictive models were checked and compared. In addition, the generalization capability of the Arrhenius type constitutive model and the ANN model was investigated under various conditions. Moreover, we propose an ML–mechanism hybrid model to improve the generalization capability of conventional constitutive models and pure data-driven models.

## 2. Experiments

A TiAl commercial alloy made by the Institute of Metal Research, Chinese Academy of Science (Shenyang, China) was utilized in this study. The nominal composition was Ti-46Al-2Cr-2Nb (at. %), and the microstructure consisted of γ-TiAl and α_2_-Ti_3_Al phases [23]. The cylindrical samples with a dimension of 15 × Φ 8 mm were compressed by a Gleeble-3800 thermomechanical simulation machine, and only axial homogeneous stresses were considered during the compression. The recommend strain rate of TiAl alloys should be smaller than 0.1 s^−1^ [11,13], and the proposed deformation conditions are listed in Table 1. Each TiAl specimen was heated from room temperature to the test temperature at a rate of 10 °C/s, held for 180 s, and then compressed to 50% true strain at the preset strain rate.

**Figure 1:**
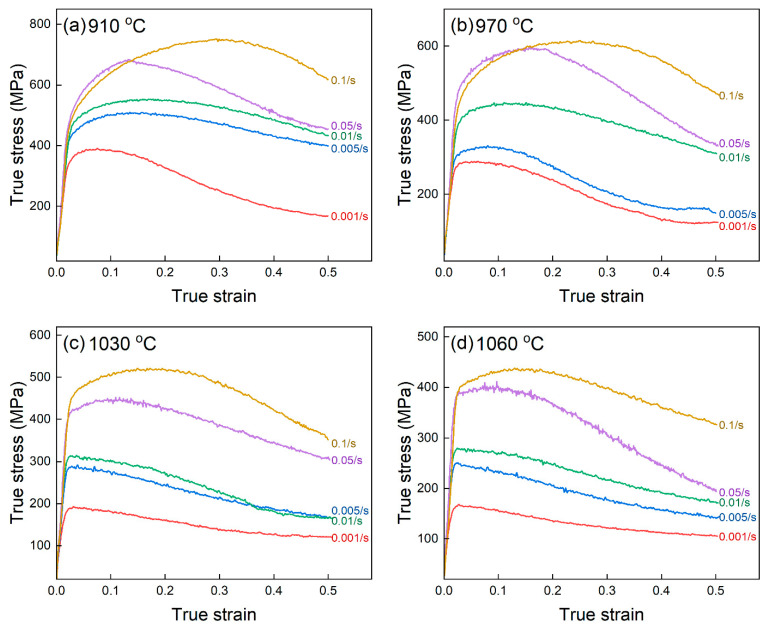
True stress–strain curves of a TiAl alloy when deformed at the elevated temperature of (**a**) 910 °C, (**b**) 970 °C, (**c**) 1030 °C, and (**d**) 1060 °C.

The true stress–strain curves obtained at different deformation conditions are given in Figure 1. The stress–strain curve macroscopically represents the competition between work hardening and softening, which can be observed in Figure 1 [24]. The work hardening phenomenon caused by dislocation multiplications was dominant at the first stage of deformations, and the flow stress increased toward the peak accordingly. Then, the work softening was more significant than the work hardening, leading to a decrease in the flow stress. The work softening mainly resulted from the dynamic recrystallization due to the low stacking fault energy in TiAl alloys [11]. The decrease in flow stress was associated with the higher deformation temperature and the slower strain rate, indicating that the flow stress was sensitive to the deformation conditions [25].

## 3. Predictive Models for Hot Deformation of TiAl Alloys

The flow stresses at eight strains of ε = 0.05, 0.1, 0.15, 0.2, 0.25, 0.3, 0.35, and 0.4 were extracted to fit three constitutive models and three ML models. The fitness of each predictive model was evaluated via the root-mean-squared error (RMSE) and the coefficient of determination (*R*^2^) expressed as follows.

(1) $$\begin{array}{c} RMSE=\sqrt{\frac{1}{n}{\left(y_{i}-{\hat{y}}_{i}\right)}^{2}} \end{array}$$

(2) $$\begin{array}{c} R^{2}=1-\frac{\sum_{i=1}^{n}{\left(y_{i}-{\hat{y}}_{i}\right)}^{2}}{\sum_{i=1}^{n}{\left(y_{i}-\bar{y}\right)}^{2}} \end{array}$$

where $\bar{y}$ is the mean of the actual value $y_{i}$, and ${\hat{y}}_{i}$ is the corresponding prediction. A greater *R*^2^ and a smaller RMSE mean a more accurate model.

### 3.1. Modified Johnson–Cook (MJC) Model

The Johnson–Cook (JC) model is widely employed in commercial finite element software to evaluate flow stresses of metals at high strain rates and various temperatures [26]. The JC model has been revised to Equation (3) considering the coupling effects of the strain (ε), strain rate ($\dot{\varepsilon }$ in s^−1^), and deformation temperature (*T* in K) on flow stresses (σ in MPa) [27,28].

(3) $$\begin{array}{c} \sigma =\left(B_{0}+B_{1}\varepsilon +B_{2}\varepsilon^{2}+B_{3}\varepsilon^{3}\right)\left(1+Cln{\dot{\varepsilon }}^{*}\right)\exp\left[\left(\lambda_{1}+\lambda_{2}ln{\dot{\varepsilon }}^{*}\right)T^{*}\right] \end{array}$$

where ${\dot{\varepsilon }}^{*}=\dot{\varepsilon }/{\dot{\varepsilon }}_{0}$ with ${\dot{\varepsilon }}_{0}$ being the reference strain rate, $T^{*}=T-T_{0}$ with $T_{0}$ being the reference temperature, and *B*_0_, *B*_1_, *B*_2_, *C*, $\lambda_{1}$, and $\lambda_{2}$ are material constants.

Here, the slowest strain rate 0.001/s and lowest deformation temperature 910 °C were assumed to be reference values. At the reference deformation conditions, Equation (3) can reduce to

(4) $$\begin{array}{c} \sigma =\left(B_{0}+B_{1}\varepsilon +B_{2}\varepsilon^{2}+B_{3}\varepsilon^{3}\right) \end{array}$$

**Figure 2:**
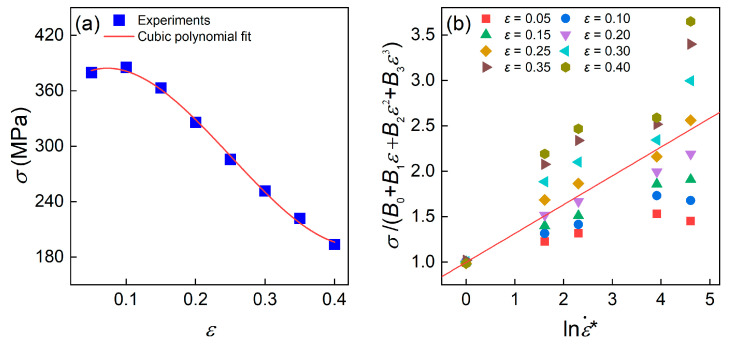
(**a**) Cubic polynomial fitting of σ and ε at the temperature of 910 °C and the strain rate of 0.001 s^−1^. (**b**) Linear relationship between $\frac{\sigma }{B_{0}+B_{1}\varepsilon +B_{2}\varepsilon^{2}+B_{3}\varepsilon^{3}}$ and $ln{\dot{\varepsilon }}^{*}$ at the temperature of 910 °C, the red line is the average fitting line.

The cubic polynomial fitting of the σ–ε plot performed in Figure 2a yielded the values of *B*_0_, *B*_1_, *B*_2_, and *B*_3_ as 357.428 MPa, 789.865 MPa, −6346.85 MPa, and 8412.79 MPa, respectively.

At the reference temperature, Equation (3) can be expressed as

(5) $$\begin{array}{c} \sigma^{\prime }=1+C\ln{\dot{\varepsilon }}^{*} \end{array}$$

In which $\sigma^{\prime }=\frac{\sigma }{B_{0}+B_{1}\varepsilon +B_{2}\varepsilon^{2}+B_{3}\varepsilon^{3}}$, and the constant *C* thus can be estimated as 0.3171 by averaging the slopes of the $\sigma^{\prime }–ln{\dot{\varepsilon }}^{*}$ plot at different strain rate, as shown in Figure 2b.

Equation (3) can also be rewritten as

(6) $$\begin{array}{c} \frac{\sigma^{\prime }}{\left(1+Cln{\dot{\varepsilon }}^{*}\right)}=\exp\left[\left(\lambda_{1}+\lambda_{2}ln{\dot{\varepsilon }}^{*}\right)T^{*}\right] \end{array}$$

Taking the natural logarithm on both sides of Equation (6) gives

(7) $$\begin{array}{c} \ln\left[\frac{\sigma^{\prime }}{\left(1+Cln{\dot{\varepsilon }}^{*}\right)}\right]=\lambda T^{*} \end{array}$$

where $\lambda =\lambda_{1}+\lambda_{2}ln{\dot{\varepsilon }}^{*}$ can be obtained by averaging the slopes of the $\ln\left[\frac{\sigma^{\prime }}{\left(1+Cln{\dot{\varepsilon }}^{*}\right)}\right]$ versus $T^{*}$ plot at various strain rates.

**Figure 3:**
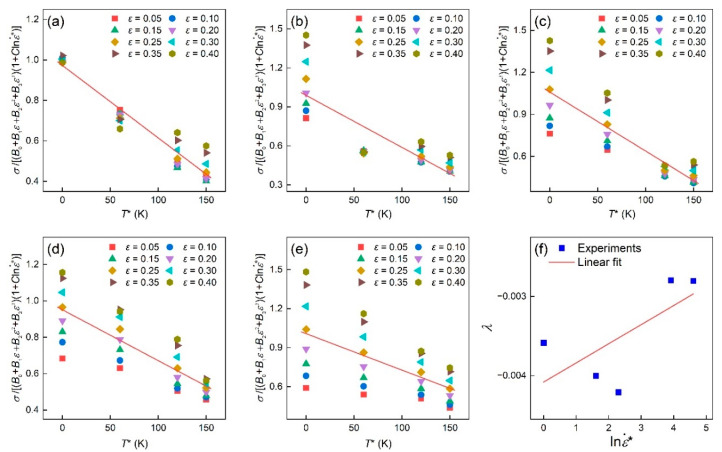
Linear relationship between $\frac{\sigma }{\left(B_{0}+B_{1}\varepsilon +B_{2}\varepsilon^{2}+B_{3}\varepsilon^{3}\right)\left(1+Cln{\dot{\varepsilon }}^{*}\right)}$ and $T^{*}$ at the strain rate of (**a**) 0.001 s^−1^, (**b**) 0.005 s^−1^, (**c**) 0.01 s^−1^, (**d**) 0.05 s^−1^, (**e**) 0.1 s^−1^, and noting that the red line is the average fitting line. (**f**) Linear relationship between  λ and $ln{\dot{\varepsilon }}^{*}$.

As shown in Figure 3a–e, the λ values were −0.00594, −0.00571, −0.00542, −0.00406, and −0.00356 when the strain rate was 0.001/s, 0.005/s, 0.01/s, 0.05/s, and 0.1/s, respectively. Next, the values of $\lambda_{1}$ and $\lambda_{2}$ can be drawn from the $\lambda –ln{\dot{\varepsilon }}^{*}$ plot in Figure 3f as −0.00408 and 0.000242, respectively. In summary, the material constants in the MJC model for the TiAl alloy are given in Table 2.

### 3.2. Modified Zerilli–Armstrong (MZA) Model

The Zerilli–Armstrong (ZA) model was proposed to describe the deformation behavior of alloys at temperatures lower than 0.6*T*_m_ (*T*_m_ is the melting temperature) [29]. Samantaray et al. [30] modified the original ZA model to predict the flow stress of metals and alloys at elevated temperatures over 0.6*T*_m_. The MZA model can be expressed as Equation (8).

(8) $$\begin{array}{c} \sigma =\left(C_{1}+C_{2}\varepsilon^{n}\right)\exp\left[-\left(C_{3}+C_{4}\varepsilon \right)T^{*}+\left[C_{5}+C_{6}T^{*}\right]\ln{\dot{\varepsilon }}^{*}\right] \end{array}$$

where *C*_1_*–C*_6_ and *n* are material constants. The reference strain rate and reference temperature are the same as the MJC model. At the reference strain rate, Equation (8) can reduce to

(9) $$\begin{array}{c} \sigma =\left(C_{1}+C_{2}\varepsilon^{n}\right)\exp\left[-\left(C_{3}+C_{4}\varepsilon \right)T^{*}\right] \end{array}$$

Taking the natural logarithm on both sides of Equation (9) gives

(10) $$\begin{array}{c} \ln\sigma =\ln\left(C_{1}+C_{2}\varepsilon^{n}\right)-\left(C_{3}+C_{4}\varepsilon \right)T^{*} \end{array}$$

**Figure 4:**
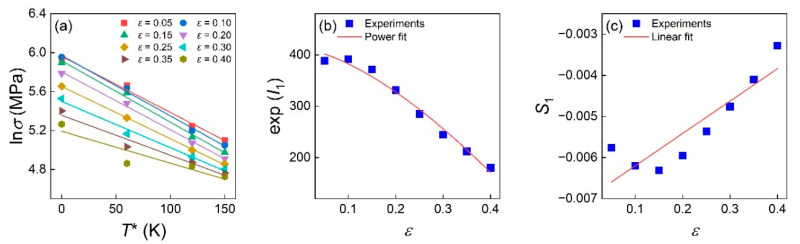
Relationship between (**a**) ln⁡σ and $T^{*}$ at various strains, (**b**) $\exp I_{1}$ and ε, and (**c**) $S_{1}$ and  ε.

As shown in Figure 4a, the intercept $I_{1}=\ln\left(C_{1}+C_{2}\varepsilon^{n}\right)$ and slope $S_{1}=-\left(C_{3}+C_{4}\varepsilon \right)$ are obtained by the linear fitting of the $\ln\sigma –T^{*}$ plot at a certain strain. Since $\exp I_{1}=C_{1}+C_{2}\varepsilon^{n}$, the power fitting of the $\exp I_{1}–\varepsilon$ plot in Figure 4b can yield the value of $C_{1}$, $C_{2}$, and *n* as 462.186, −700.252, and 1.5701, respectively. In addition, the linear fitting of the $S_{1}\;–\;\varepsilon$ plot in Figure 4c yielded the value of $C_{3}$ and $C_{4}$ as 0.00591 and 0.00013, respectively.

Taking the natural logarithms on both sides of Equation (8) yields Equation (11)

(11) $$\begin{array}{c} \ln\sigma =\ln\left(C_{1}+C_{2}\varepsilon^{n}\right)-\left(C_{3}+C_{4}\varepsilon \right)T^{*}+\left(C_{5}+C_{6}T^{*}\right)ln{\dot{\varepsilon }}^{*} \end{array}$$

**Figure 5:**
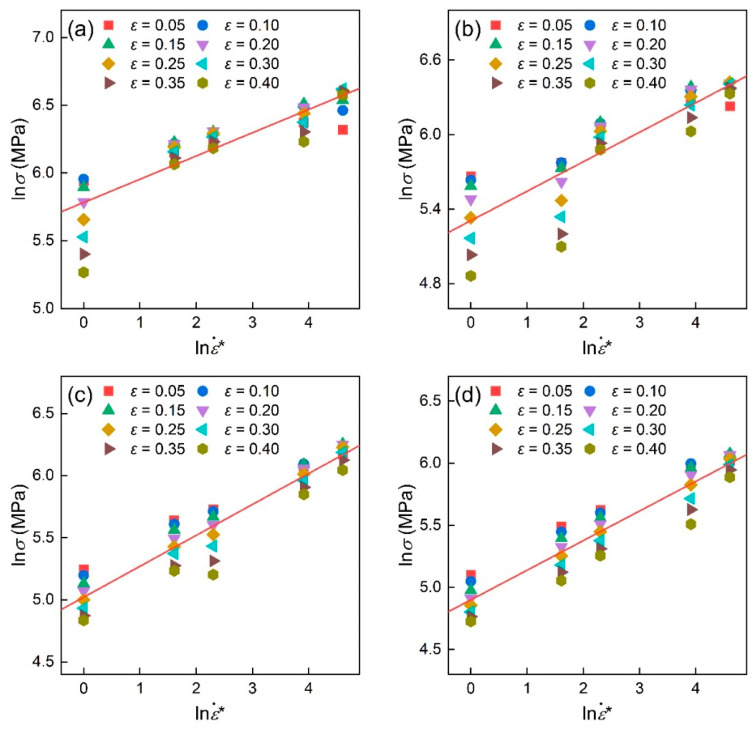
Linear relationship between ln⁡σ and $ln{\dot{\varepsilon }}^{*}$ at the temperature of (**a**) 910 °C, (**b**) 970 °C, (**c**) 1030 °C, and (**d**) 1060 °C. Noting that the red line is the average fitting line.

**Figure 6:**
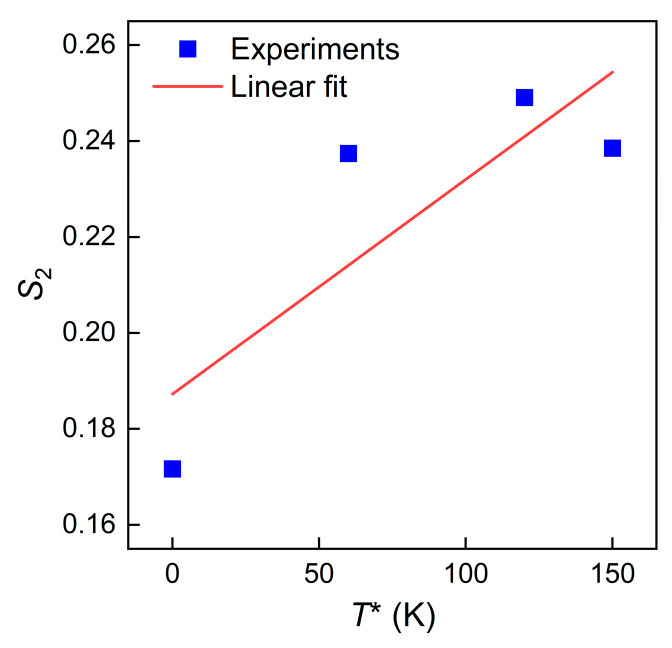
Linear relationship between $S_{2}$ and $\;T^{*}$.

At each deformation temperature, see Figure 5a–d, $S_{2}=C_{5}+C_{6}T^{*}$ was obtained by averaging the slopes of the $\ln\sigma –ln{\dot{\varepsilon }}^{*}$ plot. Then, $C_{5}$ and $C_{6}$ was calculated as 0.1580 and 0.00055 with the linear fitting of the $S_{2\;}–T^{*}$ plot shown in Figure 6. Table 3 lists the evaluated material constants of the MZA model for TiAl alloys.

### 3.3. Arrhenius Type Model

Sellars et al. [31] proposed an Arrhenius type model expressed as Equation (12) to predict the flow stress during hot deformation.

(12) $$\begin{array}{c} \dot{\varepsilon }=A{\left[\sinh\left(\alpha \sigma \right)\right]}^{n}\exp\left(-\frac{Q}{RT}\right) \end{array}$$

in which  σ is the peak stress or flow stress at a given strain ε, *R* is the universal gas constant, *α*, *A* and *n* are material constants, and *Q* is the activation energy (kJ/mol). Here, we take the peak stress as an example to demonstrate the definition of material constants *α*, *n*, *A,* and *Q.*

Taking the natural logarithm on both sides of Equation (13) gives

(13) $$\begin{array}{c} \ln\left[\sinh\left(\alpha \sigma \right)\right]=\frac{1}{n}\ln\dot{\varepsilon }-\frac{1}{n}\left(\ln A-\frac{Q}{RT}\right) \end{array}$$

**Figure 7:**
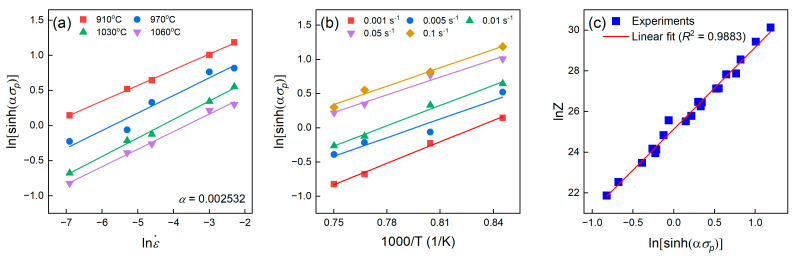
Linear relationship between (**a**) $\ln\left[\sin h\left(\alpha \sigma \right)\right]$ and $\ln\dot{\varepsilon }$, (**b**)$\;\ln\left[\sin h\left(\alpha \sigma \right)\right]$ and 1000/T, and (**c**) ln⁡Z and $\ln\left[\sin h\left(\alpha \sigma \right)\right]$ when the adjustable parameter α is 0.002532.

The value of 1/*n* is obtained by averaging the slopes of the $\ln\left[\sin h\left(\alpha \sigma \right)\right]–\ln\dot{\varepsilon }$ plot under various temperatures. In addition, *α* is an adjustable constant to ensure a linear and parallel regression of Equation (13) [31]. The present work utilized the Bayesian optimization to determine the value of *α* [22]. The average *R*^2^ value of Equation (13) reached the maximum 0.9920 when α was 0.002532. Correspondingly, n was deduced as 6.444 in Figure 7a.

Equation (13) is equivalent to Equation (14) expressed as

(14) $$\begin{array}{c} Rn\ln\left[\sinh\left(\alpha \sigma \right)\right]=Q\cdot \frac{1}{T}+R\left(\ln\dot{\varepsilon }-\ln A\right) \end{array}$$

With the adjusted α value, *Q* thus can be evaluated as the average slope of $Rn\ln\left[\sin h\left(\alpha \sigma \right)\right]\;-\;1/T$ plots at various strain rates in Figure 7b. The activation energy of the present TiAl alloy calculated by the peak stress was 319 kJ/mol, which was greater than the TiAl self-diffusion activation energy (260 kJ/mol) [32], and similar to other reported TiAl alloys (~350 kJ/mol) [33]. The addition of Cr and Nb increased the activation energy, and thus hindered the hot deformation of TiAl alloys.

With a known activation energy, the Zener–Hollomon (*Z*) parameter given as Equation (15) can describe the effect of the strain rate and temperature on the deformation behaviors.

(15) $$\begin{array}{c} Z=\dot{\varepsilon }\cdot \exp\left(\frac{Q}{RT}\right) \end{array}$$

Combining Equations (13) and (15) gives the following formula

(16) $$\begin{array}{c} \ln Z=\ln A+n\ln\left[\sinh\left(\alpha \sigma \right)\right] \end{array}$$

Thus, ln⁡A was determined as the interception of the $\ln Z–\ln\left[\sin h\left(\alpha \sigma \right)\right]$ plot, see Figure 7c. According to the definition of an inverse hyperbolic sine function, the flow stress at different strains can be predicted via Equation (17).

(17) $$\begin{array}{c} \sigma =\frac{1}{\alpha }\ln\left[{\left(\frac{Z}{A}\right)}^{\frac{1}{n}}+\sqrt{{\left(\frac{Z}{A}\right)}^{\frac{2}{n}}+1}\right] \end{array}$$

Obviously, the original Sellars model, i.e., Equation (17), ignores the influence of strain on elevated-temperature flow behaviors [34]. Since significant strain effects on flow stresses were observed in the present TiAl alloy, a strain-compensated Sellars (SCS) model should be developed. Similarity, the values of material constants *α*, *n*, *Q*, and ln*A* at eight given strains can be calculated and listed in Table 4.

**Figure 8:**
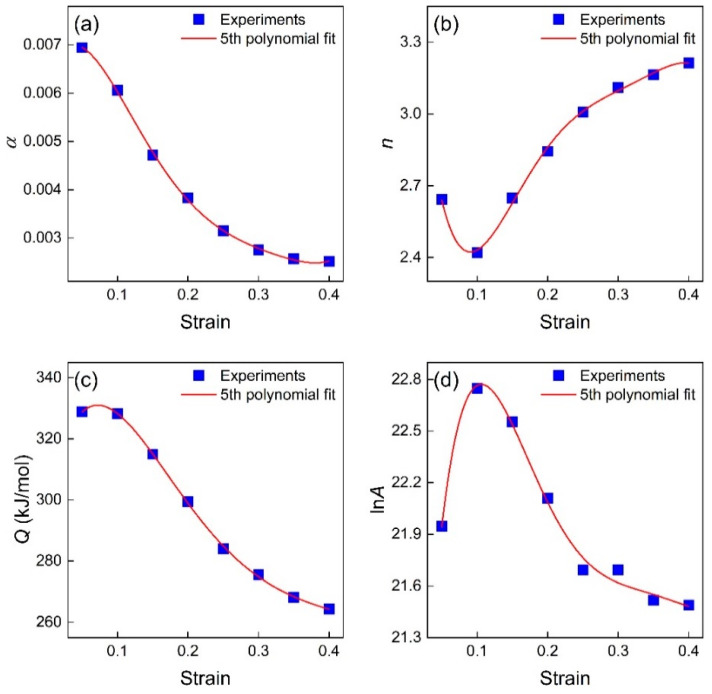
The relationship between the material constant (**a**) α, (**b**) *n*, (**c**) *Q*, and (**d**) ln*A* and the strain.

Generally, the fifth polynomial function is employed to estimate the material constant with different strains. As can be seen in Figure 8, the fifth polynomial function could fit the estimated material constants well (*R*^2^ > 0.98). Therefore, Equation (18) represents the SCS model of TiAl alloys, and the corresponding parameters are given Table 5.

(18) $$\begin{array}{c} \begin{array}{c} \sigma =\frac{1}{\alpha_{\varepsilon }}\ln\left[{\left(\frac{\dot{\varepsilon }\cdot \exp\left(\frac{Q_{\varepsilon }}{RT}\right)}{A_{\varepsilon }}\right)}^{\frac{1}{n_{\varepsilon }}}+\sqrt{{\left(\frac{\dot{\varepsilon }\cdot \exp\left(\frac{Q_{\varepsilon }}{RT}\right)}{A_{\varepsilon }}\right)}^{\frac{2}{n_{\varepsilon }}}+1}\right]\\ \alpha_{\varepsilon }=\alpha_{0}+\alpha_{1}\varepsilon +\alpha_{2}\varepsilon^{2}+\alpha_{3}\varepsilon^{3}+\alpha_{4}\varepsilon^{4}+\alpha_{5}\varepsilon^{5}\\ n_{\varepsilon }=n_{0}+n_{1}\varepsilon +n_{2}\varepsilon^{2}+n_{3}\varepsilon^{3}+n_{4}\varepsilon^{4}+n_{5}\varepsilon^{5}\\ Q_{\varepsilon }=Q_{0}+Q_{1}\varepsilon +Q_{2}\varepsilon^{2}+Q_{3}\varepsilon^{3}+Q_{4}\varepsilon^{4}+Q_{5}\varepsilon^{5}\\ {\ln A}_{\varepsilon }=A_{0}+A_{1}\varepsilon +A_{2}\varepsilon^{2}+A_{3}\varepsilon^{3}+A_{4}\varepsilon^{4}+A_{5}\varepsilon^{5} \end{array} \end{array}$$

**Figure 9:**
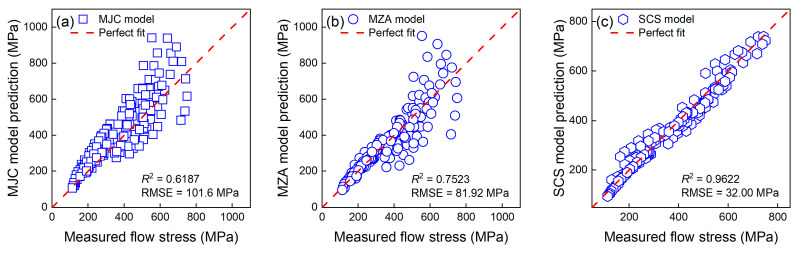
The predicted flow stress of the (**a**) MJC model, (**b**) MZA model, and (**c**) SCS model versus measured stresses.

Figure 9 compares the performance of the SCS model, MJC model, and MZA model. For more details of the three constitutive models, please see Gao et al. [35]. As can be seen, the SCS model significantly outperformed the other two constitutive models with a much greater *R*^2^ value and an RMSE smaller than 35 MPa. Hence, the SCS model is recommended for simulating the flow behavior of TiAl alloys.

### 3.4. Data-Driven Models

Since the above three constitutive models might not accurately predict the flow behaviors when the stress instability occurs, data-driven models were introduced.

Three ML models including ANN, SVR, and RF models were trained for predicting the flow stress of the considered TiAl alloys. Before constructing the ML models, a feature standardization was employed to standardize the features, i.e., the strain, strain rate, and temperature, given by

(19) $$\begin{array}{c} x^{\prime }=\frac{x\;-\;\bar{x}}{\sigma_{x}} \end{array}$$

where x and $\bar{x}$ are the actual value and average actual value of the features, respectively, and $\sigma_{x}$ is the standard deviation of the features.

There are various hyperparameters that can determine the architecture of the three aforementioned ML algorithms, listed in Table 6, which are efficient in practice [36]. We used the grid search to determine these hyperparameters based on a tenfold cross-validation. The extracted data were equally and randomly divided into ten folds. Each ML model was trained on the training set formed by nine folds and validated on the remaining one. This process was repeated ten times, and the cross-validation *R*^2^ (CV-*R*^2^) was obtained as the mean of ten validation results. The CV-*R*^2^ is regarded as an estimation of the generalization capability, and the application of a cross-validation thus can avoid overfitting.

**Figure 10:**
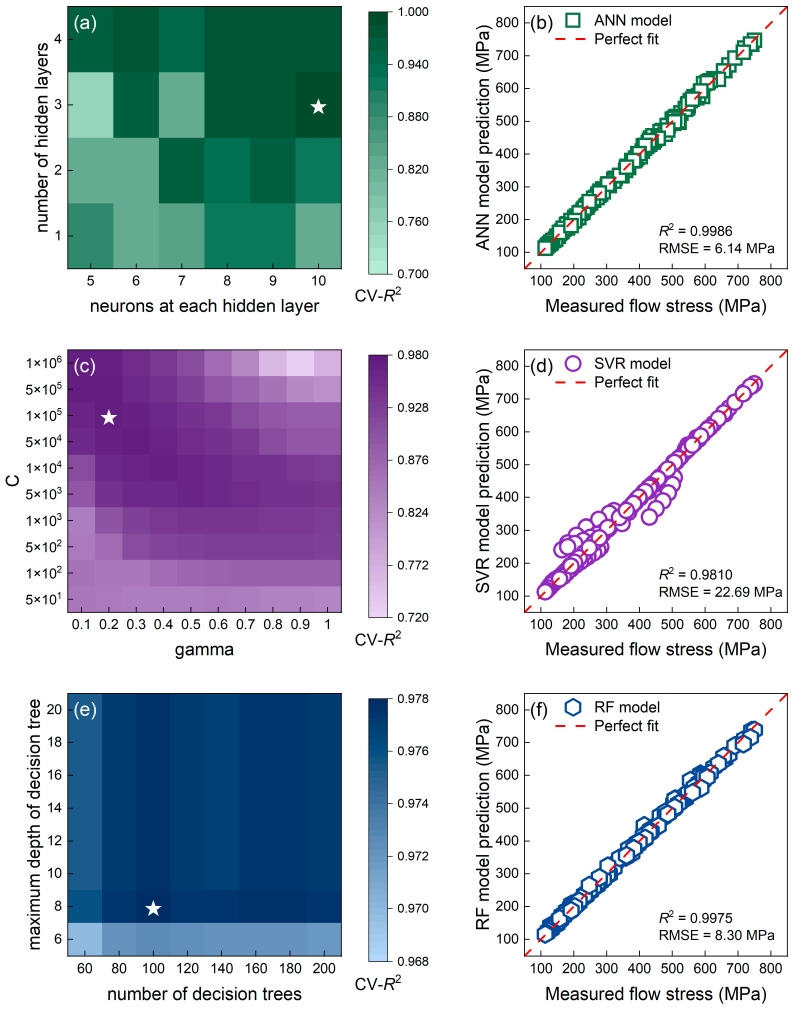
The grid search to tune hyperparameters of the (**a**) ANN model, (**c**) SVR model, and (**e**) RF model. The corresponding predicted flow stress of the (**b**) tuned ANN model, (**d**) tuned SVR model, and (**f**) tuned RF model versus the measured values.

As can be seen in Figure 10a,c,e, the cross-validated *R*^2^ (CV-*R*^2^) value of all three data-driven models reached 0.97 and higher. The ANN model had the maximum CV-*R*^2^ value of 0.9869 when it had three hidden layers, each of which contained 10 neurons (marked with the white star in Figure 10a). In addition, the CV-*R*^2^ value for the SVR model was maximized at 0.9728 when C = 100,000 and gamma = 0.2 (white star in Figure 10c), and that for the RF model was maximized at 0.9779 when max_depth = 8 and n_estimators = 100 (white star in Figure 10e). The tuned ANN model had the greatest CV-*R*^2^ value among the three ML models, indicating that it had the widest generalization capability.

Predictions of three data-driven models on eight given strains are illustrated in Figure 10b,d,f versus the measured values. All data-driven models outperformed the constitutive models with *R*^2^ value greater than 0.98. In particular, the ANN model had an excellent prediction accuracy, as evidenced by an extremely high *R*^2^ value of 0.9986.

## 4. Generalization Capability of Predictive Models

A good predictive model should have a high prediction accuracy and a wide generalization capability. Therefore, based on the prediction accuracy of three constitutive models and three ML models on training data, only the generalization capabilities of the SCS model and ANN model were further examined.

### 4.1. Generalization Capability at Interpolated and Extrapolated Strains

**Figure 11:**
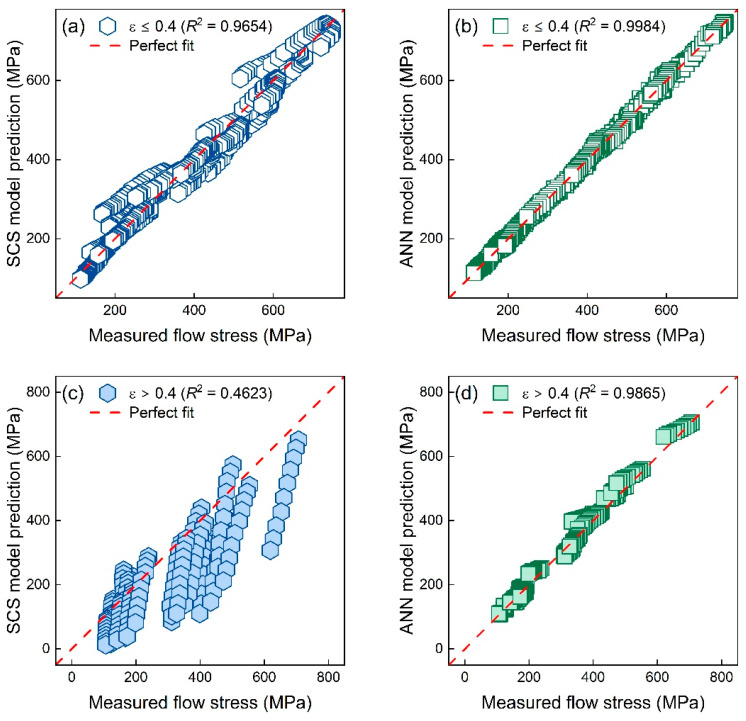
True flow stresses of TiAl alloys under given deformation conditions at interpolated strains and corresponding values predicted by the (**a**) SCS model and (**b**) ANN model. True flow stresses of TiAl alloys at extrapolated strains and corresponding values predicted by the (**c**) SCS model and (**d**) ANN model.

Figure 11a,b show the predicted stress–strain responses of the SCS model and ANN model at interpolated strains (0.05 to 0.4 with an interval of 0.01). As expected, both the constitutive model and ML model performed well at interpolated strains. The ANN model had an extremely high *R*^2^ value greater than 0.998, and the SCS model had a slightly smaller *R*^2^ value of 0.9642. However, as seen in Figure 11c,d, the accuracy of the two predictive models decreased to varying degrees at extrapolated strains (0.41 to 0.5 with an interval of 0.01). The ANN model still performed well at extrapolated strains with a high *R*^2^ value of 0.9865, while the *R*^2^ value of SCS model was only 0.4623 at extrapolated strains.

The mechanism-based SCS model significantly underestimated the flow stress of TiAl alloys at extrapolated strains. This can be explained by the fact that the deformation mechanism of TiAl alloys at large strains might be different from those at small strains, and a similar phenomenon was also reported in nickel-based superalloys [37]. Nevertheless, the pure data-driven ANN model is independent of the mechanism and thus can accurately predict the flow stress at extrapolated strains.

### 4.2. Generalization Capability at Unknown Deformation Conditions

**Figure 12:**
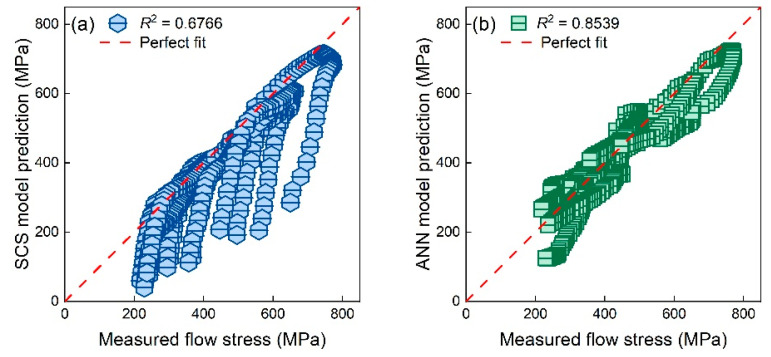
True flow stresses of TiAl alloys under new deformation conditions at strains between 0.05 and 0.5 and corresponding values predicted by the (**a**) SCS model and (**b**) ANN model.

In addition, the stress–strain response under unknown deformation conditions listed in Table 7 was also predicted by the SCS model and ANN model. Unfortunately, neither predictive models could perform well under new deformation conditions; see Figure 12. The *R*^2^ value of the SCS model and ANN model were 0.8539 and 0.6766, respectively.

As previously mentioned, the ANN model did not consider any deformation mechanism. Therefore, the accuracy of the ANN model under unknown conditions decreased by around 15% compared to that under known conditions. The underlying mechanism under different conditions was similar. Hence, the performance of the SCS model under unknown conditions was nearly the same as that under known conditions. That is, the SCS model could predict the flow stress at small strains (<0.4) well, while its performance at larger strains was extremely poor.

### 4.3. Improvement of Generalization Capability

**Figure 13:**
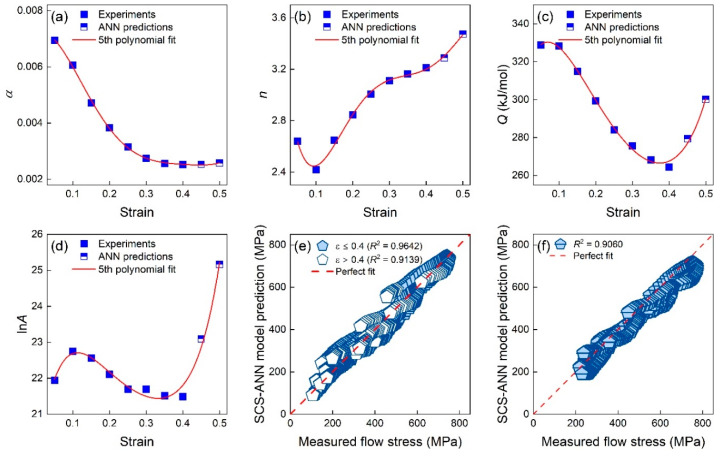
The fifth polynomial fitting of material constants (**a**) α, (**b**) *n*, (**c**) *Q*, and (**d**) ln*A* with experiments and ANN predictions for the SCS-ANN model. The predictions of the SCS-ANN model under the (**e**) given deformation conditions and (**f**) new deformation conditions.

Based on the generalization capability of the SCS model and ANN with different inputs, we proposed a mechanism–ML hybrid model. The predictions of the ANN model at large strains (0.45 and 0.5 here) were combined with the experimental results at small strain (0.05–0.4) to determine the parameters of the SCS model. The obtained SCS model based on hybrid inputs was termed as SCS-ANN model, and the fifth polynomial fits of material constants for the SCS-ANN model are shown in Figure 13. The related parameters of the SCS-ANN model are listed in Table 8.

At known deformation conditions, the *R*^2^ values of the SCS-ANN model when predicting the stress–strain response at interpolated and extrapolated strains were 0.9642 and 0.9139, respectively. The introduction of ANN predictions could significantly improve the performance of the SCS models at extrapolated strains. For new deformation conditions, the *R*^2^ value of the SCS-ANN model could reach 0.9, meaning a better generalization capability than conventional constitutive models as well as data-driven models. The hybrid SCS-ANN model is an accurate and generalized model for predicting the hot-deformation behaviors of the considered TiAl alloys.

## 5. Conclusions

This work investigated the hot-deformation behaviors of TiAl alloys under different loading strain rates at various deformation temperatures. The experimental results were analyzed by three constitutive models including the MJC model, MZA model, and SCS model. In addition, an ML-based ANN model, SVR model, and RF model were employed to predict the hot-deformation behaviors as well. The following conclusion can be summarized.

(1) The hot-deformation behaviors of TiAl alloys were examined under different deformation conditions. The flow stress of the TiAl alloys was found to be sensitive to the deformation temperature and strain rates. The activation energy of Ti-46Al-2Cr-2Nb was 319 kJ/mol, which was greater than the TiAl self-diffusion activation energy.
(2) The Arrhenius type SCS model had a better predictive accuracy than the MJC and MZA models, with an *R*^2^ value of 0.9622 on the training data. As for the generalization capability, the SCS model only performed well at interpolated strains under known deformation conditions, and the corresponding *R*^2^ value was 0.9654. The *R*^2^ value of SCS model at extrapolated strains or under new deformation conditions was smaller than 0.7.
(3) All three data-driven models performed better than the constitutive models, and the ANN model performed the best with an extremely high *R*^2^ value of 0.9986. The ANN model had a good generalization capability under known deformation conditions; the *R*^2^ values at interpolated strains and extrapolated strains were 0.9984 and 0.9865, respectively. However, the ANN model could not perform well under new deformation conditions, with a corresponding *R*^2^ value of only 0.8539.
(4) For limited hot-deformation experimental data, the ANN model was recommended to predict the flow behavior of TiAl alloys at larger strains. Then, predictions of the ANN model were further combined with experimental results to construct an ML–mechanism hybrid SCS-ANN model. The hybrid model was more accurate than the pure data-driven model and mechanism-based model under unknown deformation conditions.

## Figures and Tables

**Table 1:** The deformation conditions performed on the Gleeble-3800.

Deformation Temperature (°C)	Strain Rate (s^−1^)
910	0.001, 0.005, 0.01, 0.05, 0.1
970	0.001, 0.005, 0.01, 0.05, 0.1
1030	0.001, 0.005, 0.01, 0.05, 0.1
1060	0.001, 0.005, 0.01, 0.05, 0.1

**Table 2:** Fitting material constants in the MJC model for the TiAl alloy.

$B_{0}$ (MPa)	$B_{1}$ (MPa)	$B_{2}$ (MPa)	$B_{3}$ (MPa)	C	$\lambda_{1}$	$\lambda_{2}$
357.428	789.865	−6346.85	8412.79	0.3171	−0.00408	0.000242

**Table 3:** Fitting material constants in the MZA model for the TiAl alloy.

$C_{1}$ (MPa)	$C_{2}$ (MPa)	*n*	$C_{3}$	$C_{4}$	$C_{5}$	$C_{6}$
462.186	−700.252	1.5701	0.00591	0.00013	0.1580	0.000551

**Table 4:** The value of material constants of *α* (MPa^−1^), *n*, *Q* (kJ/mol), and ln*A* (s^−1^) for TiAl alloys at different strains.

	Strain							
	0.05	0.1	0.15	0.2	0.25	0.3	0.35	0.4
*α*	0.00694	0.00606	0.00472	0.00383	0.00315	0.00275	0.00257	0.00252
*n*	2.6420	2.4198	2.6484	2.8438	3.0079	3.1105	3.1633	3.2133
*Q*	328.78	328.24	314.93	299.42	284.08	275.56	268.11	264.35
ln*A*	21.946	22.750	22.554	22.109	21.692	21.692	21.516	21.489

**Table 5:** The coefficients of the polynomial fitting of material constants.

$\alpha_{\varepsilon }$ (MPa^−1^)	$n_{\varepsilon }$	$Q_{\varepsilon }$ (kJ/mol)	${\ln A}_{\varepsilon }$ (s^−1^)
$\alpha_{0}=0.0063$	$n_{0}=3.9754$	$Q_{0}=300.92$	$A_{0}=18.613$
$\alpha_{1}=0.0412$	$n_{1}=-44.982$	$Q_{1}=979.24$	$A_{1}=105.21$
$\alpha_{2}=-0.7224$	$n_{2}=452.30$	$Q_{2}=-10089$	$A_{2}=-936.46$
$\alpha_{3}=3.5140$	$n_{3}=-1919.6$	$Q_{3}=35579$	$A_{3}=3591.7$
$\alpha_{4}=-7.4003$	$n_{4}=3787.0$	$Q_{4}=-56055$	$A_{4}=-6397.1$
$\alpha_{5}=5.8484$	$n_{5}=-2854.9$	$Q_{5}=33581$	$A_{5}=4347.1$

**Table 6:** The hyperparameters of three data-driven models.

Model	Hyperparameters	Description
ANN model	hidden_layers_sizes	10 means a single hidden layer with 10 neurons 8,8 means 2 hidden layers with 8 neurons at each hidden layer
SVR model	C	Penalty parameter for the regularization
	gamma	Gamma value of the radial basis function kernel function
RF model	max_depth	Maximum depth of each decision tree
	n_estimators	Number of decision trees in the RF model

**Table 7:** The new deformation conditions to examine generalization capabilities.

Deformation Temperature (°C)	Strain Rate (s^−1^)
940	0.001, 0.005, 0.01, 0.05
910, 970, 1030, 1060	0.025
910, 970, 1030, 1060	0.075

**Table 8:** The coefficients of material constants based on experiments and ANN predictions.

$\alpha_{\varepsilon }$ (MPa^−1^)	$n_{\varepsilon }$	$Q_{\varepsilon }$ (kJ/mol)	${\ln A}_{\varepsilon }$ (s^−1^)
$\alpha_{0}=0.0070$	$n_{0}=3.6195$	$Q_{0}=308.23$	$A_{0}=19.506$
$\alpha_{1}=0.0138$	$n_{1}=-31.751$	$Q_{1}=737.87$	$A_{1}=74.980$
$\alpha_{2}=-0.3831$	$n_{2}=289.51$	$Q_{2}=-7635.9$	$A_{2}=-615.03$
$\alpha_{3}=1.6848$	$n_{3}=-1050.2$	$Q_{3}=25689$	$A_{3}=2189.3$
$\alpha_{4}=-2.9723$	$n_{4}=1703.3$	$Q_{4}=-40648$	$A_{4}=-3847.3$
$\alpha_{5}=1.9065$	$n_{5}=-1018.7$	$Q_{5}=27578$	$A_{5}=2840.9$

## Data Availability

The data presented in this study are available on request from the corresponding author.
